# From Fabrication to Function: Scalable Spray-Assisted Layer-by-Layer Films for Drug Delivery in Glaucoma

**DOI:** 10.3390/ijms27188257

**Published:** 2026-09-16

**Authors:** Alexandra M. L. Oliveira, Maria Raposo, Sorawee Yanwinitchai, Robert O. Williams, Nykola C. Jones, Søren V. Hoffmann, Marta P. Nascimento, Mafalda Guerreiro, Diogo B. Bitoque, Mariana M. Silva, Luís Abegão Pinto, Quirina Ferreira, Gabriela A. Silva

**Affiliations:** 1 NOVA Medical School, Faculdade de Ciências Médicas (NMS, FCM), Universidade Nova de Lisboa, 1160-056 Lisboa, Portugal; marta.nascimento@nms.unl.pt (M.P.N.); mafaldaguerreiroo@gmail.com (M.G.); diogo.bitoque@nms.unl.pt (D.B.B.); marianams2003@gmail.com (M.M.S.); gabriela.silva@nms.unl.pt (G.A.S.); 2 iNOVA4Health, NOVA Medical School, Faculdade de Ciências Médicas (NMS, FCM), Universidade Nova de Lisboa, 1160-056 Lisboa, Portugal; 3 Laboratory for Instrumentation, Biomedical Engineering and Radiation Physics (LIBPhys-UNL), Associated Laboratory in Translation and Innovation Towards Global Health (LA-REAL), Department of Physics, NOVA School of Science and Technology, NOVA University of Lisbon, Campus FCT-UNL, 2829-516 Caparica, Portugal; 4 Division of Molecular Pharmaceutics and Drug Delivery, College of Pharmacy, The University of Texas at Austin, Austin, TX 78712, USA; s.yanwinitchai@utexas.edu (S.Y.); bill.williams@austin.utexas.edu (R.O.W.III); 5 ISA, Department of Physics and Astronomy, Aarhus University, Ny Munkegade 120, 8000 Aarhus, Denmark; nykj@phys.au.dk (N.C.J.); vronning@phys.au.dk (S.V.H.); 6 Ophthalmology Department, Centro Hospitalar Universitário de Lisboa Norte, 1649-035 Lisboa, Portugal; abegaopinto@gmail.com; 7 VisionLab, Lisbon School of Medicine, University of Lisbon, 1649-028 Lisboa, Portugal; quirinatf@gmail.com; 8 Escola Superior de Saúde de Lisboa, Polytechnic University of Lisbon, 1990-096 Lisboa, Portugal

**Keywords:** glaucoma, drug delivery system, layer-by-layer, 5-fluorouracil, β-cyclodextrin, graphene oxide

## Abstract

Glaucoma drainage device (GDD) surgery is frequently limited by postoperative fibroblast proliferation, leading to device failure and the need for revision procedures. In this work, we developed a spray-assisted layer-by-layer (LbL) drug delivery system for GDD surfaces intended to enable localized release of antiproliferative agents, as a proof-of-concept approach toward more standardized patient care. Multilayer thin films composed of poly(β-amino ester) (PBAE) and a 5-fluorouracil (5-FU) and β-cyclodextrin (β-CD) complex (5-FU:β-CD) were fabricated, without (type A films) and with (type B films) graphene oxide (GO) barrier layers. Film growth was monitored by ultraviolet–visible spectroscopy (UV-Vis) and vacuum ultraviolet spectroscopy (VUV), confirming consistent, sequential deposition; type A films exhibited lower variability than GO-containing type B films. Drug release studies revealed a rapid burst release within the first 5 min, followed by a plateau, regardless of GO incorporation, indicating that sustained drug release was not achieved under the current film architecture. Cell cycle analysis confirmed that β-CD complexation preserved the biological activity of 5-FU, which induced an S-phase arrest in a dose-dependent manner. Although further optimization of film architecture and composition is required to improve release control and achieve sustained delivery, this study demonstrates the feasibility of spray-assisted LbL coatings as a localized drug delivery strategy for GDD surfaces. Overall, these findings support the proof-of-concept nature of this approach and identify key parameters that must be optimized before translational applicability can be established.

## 1. Introduction

Affecting an estimated 76 million people worldwide in 2020, glaucoma is one of the leading causes of blindness [1]. It is a severe neurodegenerative disease characterized by the loss of retinal ganglion cells and progressive optic nerve atrophy [2,3,4,5]. Although glaucoma is normally associated with intraocular pressure (IOP), the underlying mechanisms remain incompletely understood, and the disease can also occur in the absence of increased IOP [2,6].

The IOP rises in response to failure of the aqueous humor drainage pathways. Aqueous humor is constantly being produced inside the eye and being drained through Schlemm’s canal and/or the trabecular meshwork [6]. For primary glaucoma, we can classify the disease into two main groups: primary open-angle glaucoma (POAG) or primary angle-closure glaucoma (PACG) [7]. In POAG, the angle between the iris and the trabecular meshwork is open; the trabecular meshwork is mainly affected, and Schlemm’s canal may be functional. In PACG, the iris is enlarged, causing closure of the drainage angle (which can be total or partial) and impeding the correct drainage through Schlemm’s canal and the trabecular meshwork [7,8,9]. In this case, even though in the beginning the trabecular meshwork and Schlemm’s canal may be functional, this blockage caused by the iris will ultimately lead to permanent damage [8]. Furthermore, glaucoma can also be congenital, normally caused by an abnormal development of the anterior chamber during embryogenesis or secondary to other pathologies or syndromes, such as in the cases of juvenile glaucoma (often genetic), neovascular glaucoma, uveitic glaucoma, steroid-induced glaucoma, lens-induced glaucoma, or trauma-induced glaucoma [10,11,12,13,14,15,16,17].

Current pharmacological management of glaucoma primarily relies on topical IOP-lowering agents. β-Adrenergic blockers and carbonic anhydrase inhibitors mainly reduce aqueous humor production, while α2-adrenergic agonists decrease aqueous humor production and may also enhance uveoscleral outflow. Prostaglandin analogues predominantly increase uveoscleral outflow, whereas cholinergic agonists and Rho-associated protein kinase (ROCK) inhibitors enhance aqueous humor drainage mainly through the trabecular pathway [18,19,20]. In parallel with these IOP-lowering strategies, novel therapeutic approaches targeting retinal neuroprotection have also been explored. Among these, neuroprotective steroids such as 17β-estradiol and dehydroepiandrosterone sulfate (DHEA-S) have demonstrated protective effects against retinal injury in preclinical models, potentially involving sigma-1 receptor signaling [21]. Additionally, 17β-estradiol has demonstrated retinal ganglion cell protection and preservation of visual function in experimental models of glaucoma [22,23]. More recently, the retina-targeted prodrug 10β,17β-dihydroxyestra-1,4-dien-3-one (DHED), which selectively delivers 17β-estradiol to the retina, has also shown retinal ganglion cell protection and preservation of visual function in an experimental model of glaucoma [24]. However, this type of treatment does not always work because the drops are not effective or due to poor patient compliance [25,26,27,28]. When this happens, the next step is usually surgery [9]. There is a wide range of glaucoma surgical procedures, including laser procedures, such as laser trabeculoplasty or cyclophotocoagulation; filtering surgeries such as trabeculectomy; glaucoma drainage devices (GDDs) such as Ahmed or Paul valves; minimally invasive glaucoma surgery (MIGS), such as iStent, among others [29,30,31,32,33,34,35,36]. In this study, we focus on GDD surgery, a common approach for refractory glaucoma. Despite being widely used and considered an effective approach for glaucoma management, this procedure, like any surgical technique, has inherent risks and limitations [32]. Surgical failure often occurs due to excessive fibroblast proliferation during the wound-healing process, which can lead to obstruction of the device and subsequent loss of function [37,38,39]. As a result, a substantial proportion of patients require a return to the operating room for revision surgery [40]. Nowadays, surgeons intraoperatively apply mitomycin-C (MMC) soaked sponges, which are applied under the conjunctiva at the implantation site for a period of time determined by the surgeon (normally ranging from 1.5 to 2 min) at concentrations ranging from 0.2 to 0.4 mg/mL [41,42]. Before this, it was also common to use 5-fluorouracil (5-FU) post-surgery by subconjunctival injection daily for a week or more to maintain fibroblast suppression [41,42,43]. However, the epithelial toxicity of 5-FU, together with the discomfort associated with repeated injections, has led to a decline in its clinical use [44,45]. Notwithstanding, both MMC and 5-FU are antimitotic drugs with proven effects on cell cycle arrest and preventing excessive fibroblast proliferation [46,47]. These drugs act in different ways: MMC is an alkylating agent that crosslinks DNA replication and transcription leading to the cell cycle arrest and apoptosis in a cell-cycle-phase-independent manner; 5-FU is an antimetabolite/pyrimidine analog that is metabolized inside the cells into fluorodeoxyuridine monophosphate, which inhibits thymidylate synthase, an enzyme needed for DNA synthesis. Therefore, 5-FU is S-phase specific, only affecting cells that are actively dividing [48,49].

There is no universal standard for MMC use, and its application varies depending on the surgeon performing the surgery. The concentration, exposure time, and even the way sponges are placed all depend on the surgeon’s training, patient profile, and institutional habits, making it an operator-dependent and heterogeneous technique [50,51].

**Figure 1:**
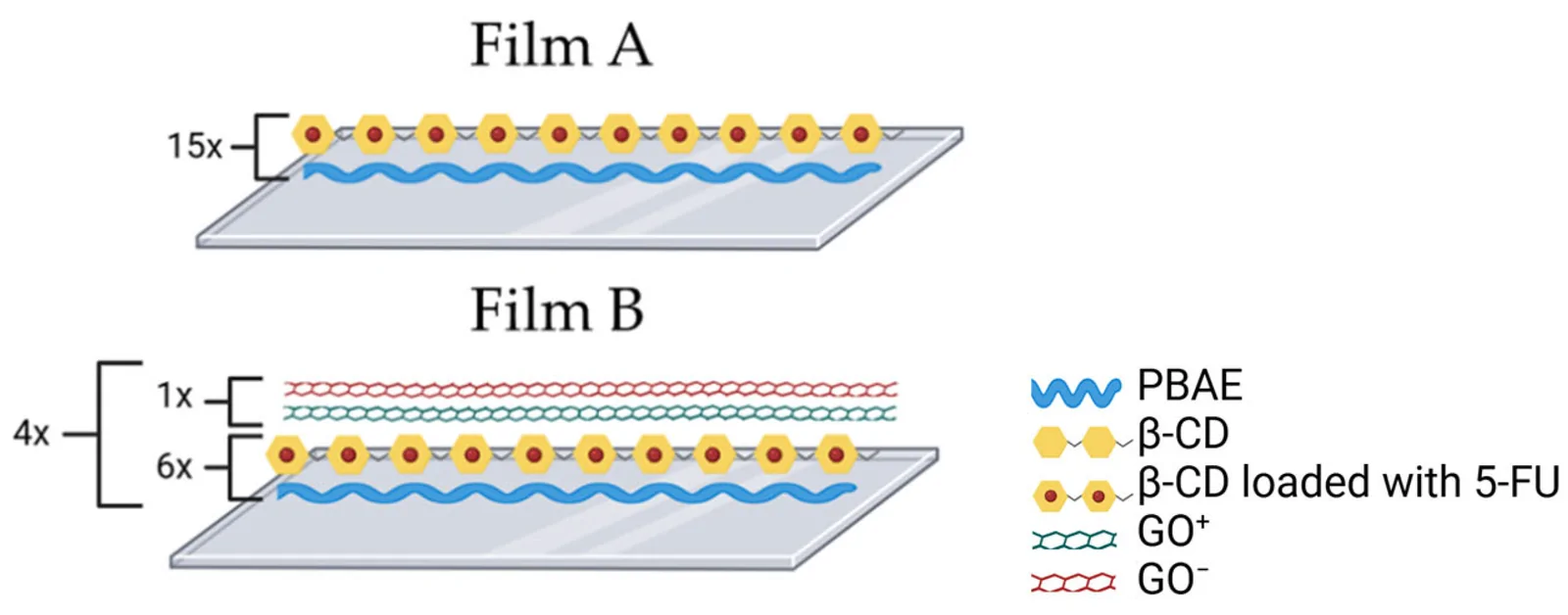
Schematic composition of type A and B LbL films. Created with BioRender.com.

We propose to address this challenge by designing a nanometer-scale drug delivery system (DDS) that can be applied to the valve plate, aiming to improve localized delivery of antiproliferative agents. As proof of concept, a multilayer DDS was developed using a spray-assisted layer-by-layer (LbL) assembly technique, in which successive layers are adsorbed onto preceding ones through electrostatic interactions, hydrogen bonding, or π–π stacking/hydrophobic interactions [52,53]. The thin films that constitute the DDS are composed of alternating layers of poly β-amino ester (PBAE) (Figure 1), a hydrophobic polymer with a backbone that is hydrolyzed under physiological conditions, allowing the drug to be released, 5-FU:β-cyclodextrin complex (5-FU:β-CD) (Film type A) and, depending on the film, they can have additional graphene oxide (GO) barrier or capping layers, either positively or negatively charged (GO^+^/GO^−,^ respectively) (Film type B) [53,54,55].

The selected architectures, (PBAE/5-FU:β-CD)_15_ for type A films and ((PBAE/5-FU:β-CD)_6_/GO^+^/GO^−^)_4_ for type B films, were based on preliminary deposition experiments and previous LbL systems developed by the group (unpublished data). In these preliminary tests, increasing the number of deposited layers beyond a certain point did not result in proportional film growth, suggesting saturation or limited incorporation efficiency. Therefore, the type A architecture was selected to provide a measurable drug-loaded multilayer structure without excessive deposition, whereas the type B architecture was designed to evaluate whether periodically inserted GO^+^/GO^−^ blocks could act as barrier layers and modulate film desorption/release. These architectures were thus selected as proof-of-concept formulations rather than fully optimized final coating designs.

Due to its highly hydrophilic nature, small size, and highly polar nature, free 5-FU was not efficiently incorporated into the film’s hydrophobic PBAE-based multilayer system. Due to PBAE’s incorporation after drying, the films have a hydrophobic nature, making it harder for 5-FU to be retained in the film since the abundance of hydrophobic interactions can repel small, highly polar molecules. Preliminary deposition attempts using non-complexed 5-FU did not result in measurable sequential film growth. Therefore, 5-FU was complexed with β-cyclodextrin (β-CD) to improve its incorporation into the LbL system, forming the complex 5-FU:β-CD [55,56,57,58]. This complexation has been previously reported by our group and physicochemically characterized in the literature, including spectroscopic and thermal analyses [58]. In the present study, the same complexation strategy (1:1 molar ratio and 24 h of stirring) was adopted to enable film growth. Successful film assembly with 5-FU: β-CD, in contrast to free 5-FU, supports the practical role of β-CD in enabling 5-FU incorporation into the multilayer system. β-cyclodextrin is a cyclic oligosaccharide composed of seven glucose units linked by α-1,4 glycosidic bonds, and its conical shape creates an outer hydrophilic surface with an inner hydrophobic cavity, which makes it ideal for drug complexation, improving solubility, stability, and bioavailability. β-CD was preferred over α- or γ-cyclodextrins because the cavity size, which is about 6.0–6.5 Å, makes it ideal to complex with 5-FU, which has a size of about 5–6 Å, and it has been widely reported in the literature to form stable complexes with several drugs [57,59].

Graphene is a two-dimensional compound composed of a monolayer of aromatic carbon atoms (sp^2^-hybridized), covalently bound in a hexagonal lattice structure, forming sheets only one atom thick. Its oxidized form, GO, retains this sheet-like architecture while introducing abundant oxygen-containing functional groups (hydroxyl, epoxy, carboxyl). When used as a capping or barrier layer in multilayer films, GO provides a dense, planar barrier that slows molecular diffusion by creating a tortuous pathway. This barrier effect enhances film stability and regulates drug release [53].

In this work, considering our proposed DDS development strategy, the growth of the proposed type A and type B films was characterized by ultraviolet-visible (UV-Vis) and vacuum ultraviolet (VUV) spectroscopies; 5-FU release/desorption was evaluated by high-performance liquid chromatography (HPLC), UV-Vis, and VUV spectroscopies and the biological effect of the released drug was assessed through cell cycle analysis to investigate its impact on cell cycle progression and arrest.

## 2. Results

### 2.1. Film Growth

**Figure 2:**
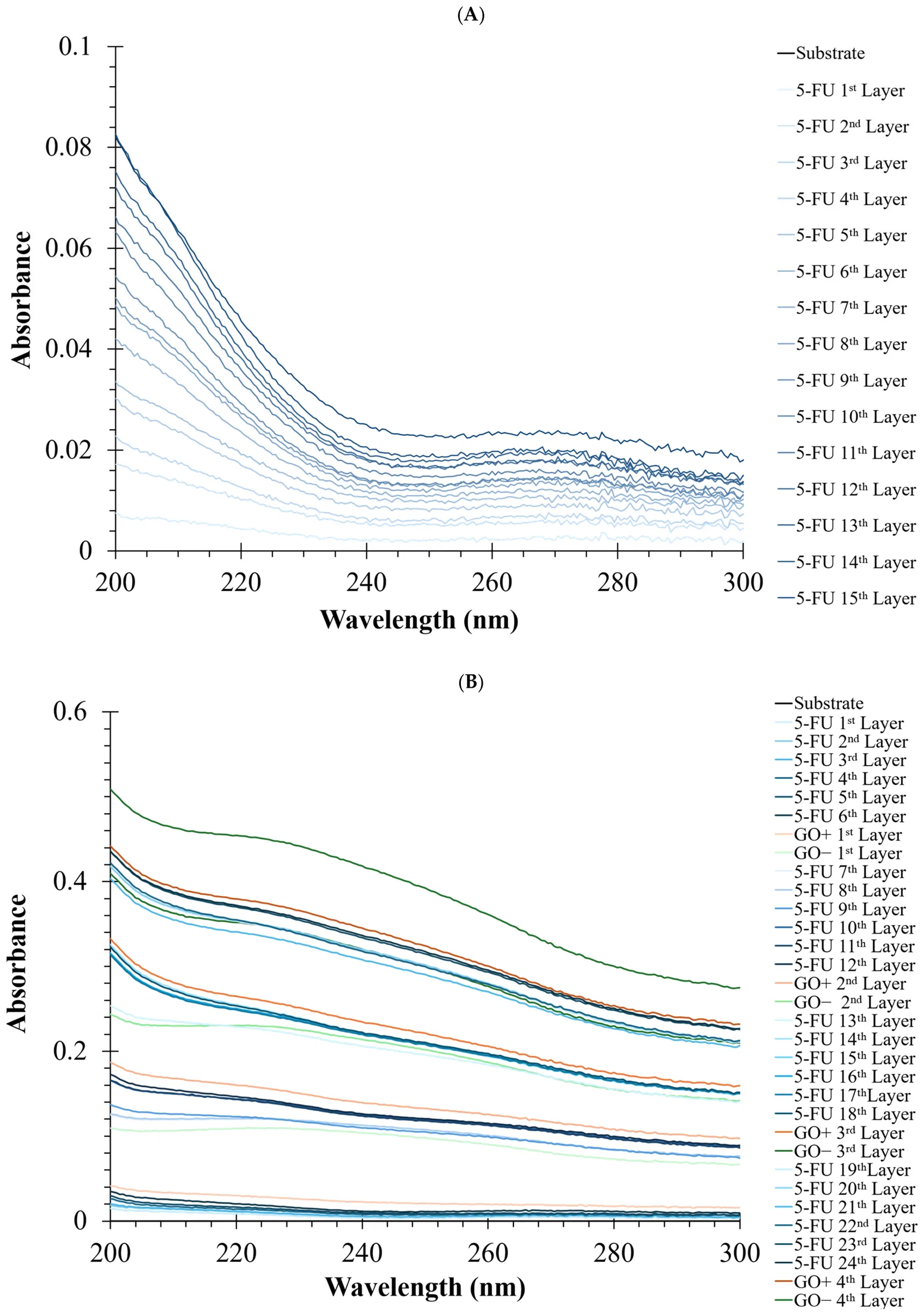
UV-Vis spectra between 200 and 300 nm representing the growth of (**A**)—(PBAE/5-FU:β-CD)_15_ (type A film) and (**B**)—((PBAE/5-FU:β-CD)_6_/GO^+^/GO^−^)_4_ (type B film) LbL films.

Films were prepared according to the protocol described in the materials and methods section of this paper. The UV-Vis absorbance spectra of LbL with A and B structures are presented in Figure 2A,B, respectively. For better visualization, only absorbance data in the 200–300 nm range are shown, as no relevant absorption occurs beyond this interval. Furthermore, at the beginning of the preparation of each film, a spectrum from the substrate was acquired, then subtracted from the following measurements for baseline correction.

As shown in Figure 2A,B, the films grow sequentially with the number of bilayers. For the films containing GO, the adsorption profile slightly changes because graphene also absorbs around 230 nm.

To infer the linearity of the absorbance with the number of bilayers, the absorbance at 266 nm was chosen as a reference for linear representation since it is the characteristic absorption wavelength for 5-FU. Moreover, β-CD does not absorb in this wavelength range, so it is not expected to change the absorption profile.

**Figure 3:**
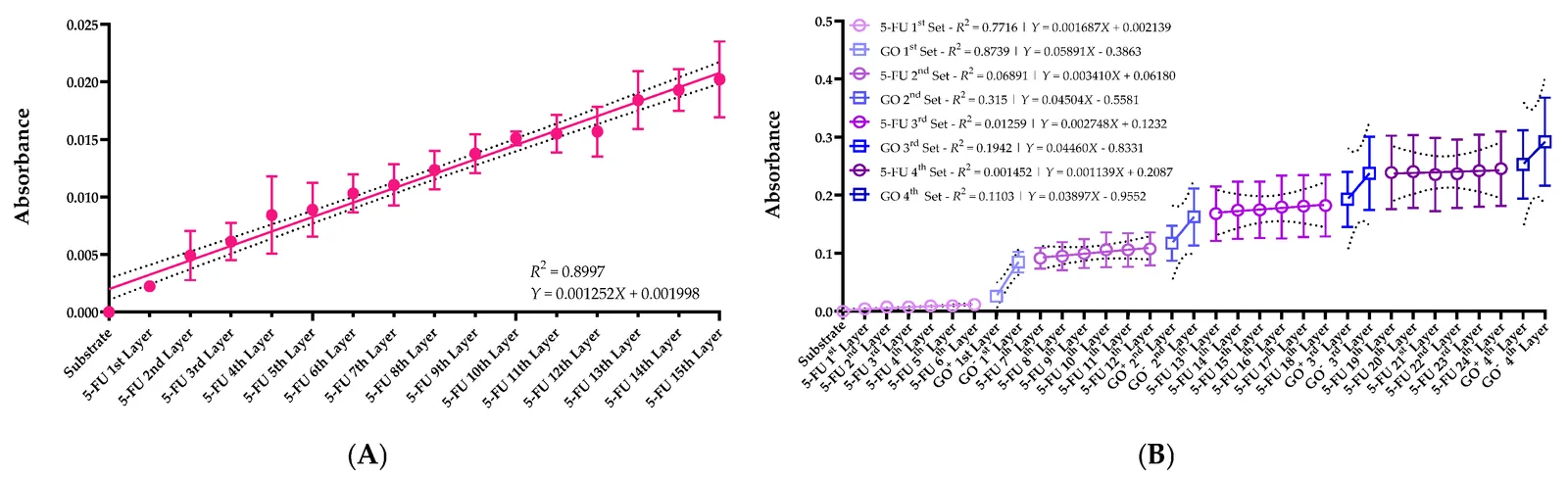

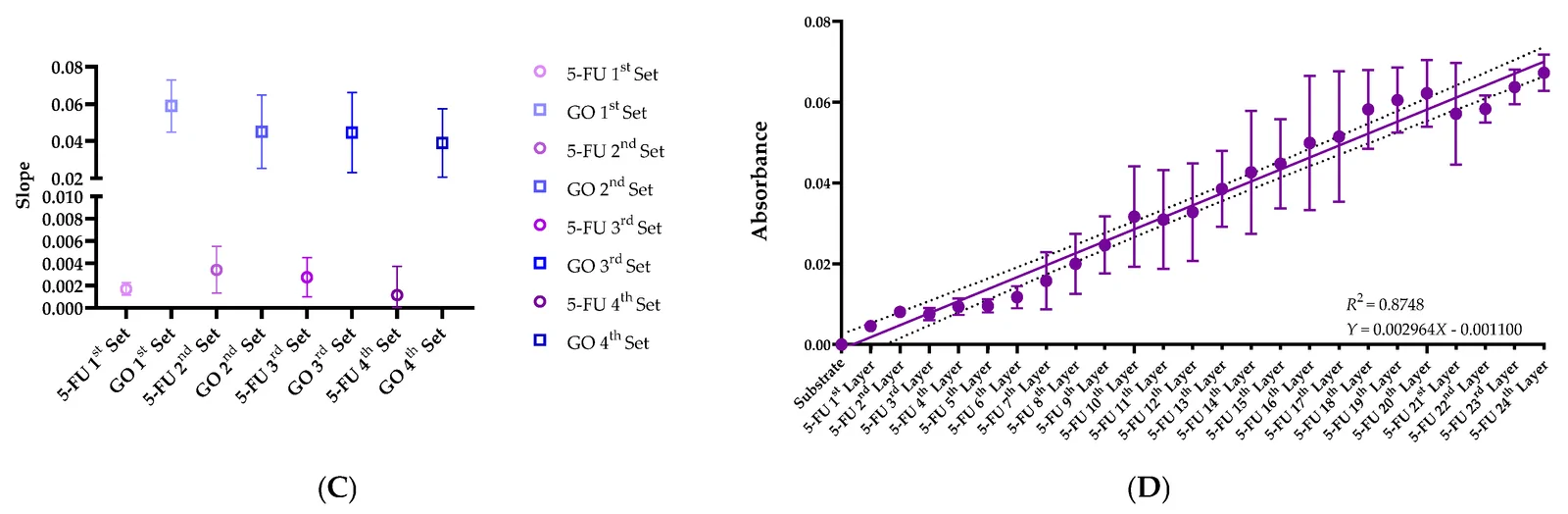
Film growth absorbance at λ 266 nm. (**A**)—(PBAE/5-FU:β-CD)_15_ (type A film); (**B**)—((PBAE/5-FU:β-CD)_6_/GO^+^/GO^−^)_4_ (type B film); (**C**)—Slope comparison per set for type B films represented in Figure 3B; (**D**)—Offset-corrected growth curve for Type B films represented in Figure 3B, isolating 5-FU accumulation from GO absorbance. Data are represented as mean ± standard deviation (SD) (*n* = 4 for Type A; *n* = 3 for Type B; see text for details). Dotted lines represent the 95% confidence interval (CI) for the regression line.

Figure 3A,B show the growth of (PBAE/5-FU:β-CD)_15_ (type A) and ((PBAE/5-FU:β-CD)_6_/GO^+^/GO^−^)_4_ (type B) LbL films, respectively, as a function of the number of bilayers. For each film type, the complete fabrication process was repeated independently, with four independent type A films and three independent type B films prepared and analyzed. These replicate numbers were used to provide a preliminary estimate of inter-film variability at this proof-of-concept stage, rather than a definitive validation of process reproducibility. Because each independent preparation required complete multilayer deposition and sequential spectroscopic monitoring, the resulting variability analysis should be interpreted as descriptive. Larger batches prepared under more standardized deposition conditions will be required to robustly assess reproducibility in future optimization studies. At each bilayer number, the absorbance values obtained from the independent films were averaged, and the error bars represent the corresponding standard deviation. For films containing GO, the growth profiles showed lower linearity, and the slopes of each set of PBAE/5-FU:β-CD bilayers were considerably lower, indicating less regular film growth and an overall smaller increase in drug loading (Figure 3C). In later layers, the variance increases, which may be expected for this type of film deposition, as the use of a manual spray atomizer and manual drying can introduce inconsistencies in layer thickness, the amount of material deposited, and overall film roughness. Aside from that, it is possible that interlayer diffusion affects the films or that each layer does not fully cover the previous one, leading to variations in the measurements that accumulate with each deposition. However, these remain possible explanations, as direct measurements of film thickness, surface morphology, roughness, or layer coverage were not performed in the present study. Because graphene layers contribute strongly to absorbance, raw growth curves show sharp step-like increases in absorbance at each GO^+^/GO^−^ insertion. To isolate the incremental growth of 5-FU, we applied an offset correction: at each graphene insertion, the cumulative offset (difference between the GO^−^ absorbance and the last preceding 5-FU value) was calculated, and this offset was subtracted from all subsequent absorbance values. The corrected curve (Figure 3D), therefore, reflects the continuous accumulation of 5-FU independently of graphene and, as indicated by the *R*^2^ value, exhibits a linear increase. The raw growth curve (Figure 3B) and the block increments reported in Table 1 provide an internal consistency check of the PBAE/5-FU:β-CD growth contribution.

Taking the data of an individual type B film as an example (see Table 1), when looking at the increments of absorbance (*A*) in each block of 6 bilayers of PBAE/5-FU:β-CD, calculated by Equation (1)

Δ*A* = *A_n_* − *A_n_*_−5_, (1)

where Δ*A* corresponds to the difference in absorbance between a set of layers; *n* represents the last bilayer number of each set. It is possible to see that the growth is similar in each block, with a mean value of 0.01338 and an SD of 0.003857, i.e., 0.013 ± 0.004.

For comparison between films, the mean area under the curve (AUC) for each film type was calculated. The relative standard deviation (RSD) or coefficient of variation (CV), given by

RSD (%) = 100 × SD/Mean(2)

was also determined for each type of film and listed in Table 2.

For type A films, in which the mean and SD values were 0.1722 and 0.01650, respectively (i.e., 0.172 ± 0.0165), the RSD value is 9.6%, indicating low relative variability of the films under the tested conditions [60,61]. For type B films, in which the mean and SD values were 4.3773 and 1.117, respectively (i.e., 4.377 ± 1.117), the RSD for these films is 25.5%. This indicates that films containing graphene exhibit greater variability than those without it [53]. However, because the raw AUC of type B films includes the strong absorbance contribution of GO, it should not be interpreted as a direct quantitative comparison of 5-FU accumulation between film types. When the AUC of the curves corrected for the GO offset was considered, accounting primarily for the drug-loaded layer increments, the mean and the SD values were 0.8281 and 0.137819, respectively, lowering the RSD value to 16.6%, suggesting improved relative consistency after removing the graphene-related background contribution. This corrected value indicates moderate variability and is comparable to RSD values reported for spray-based coating processes [62,63,64].

### 2.2. Film Desorption/Drug Release

**Figure 4:**
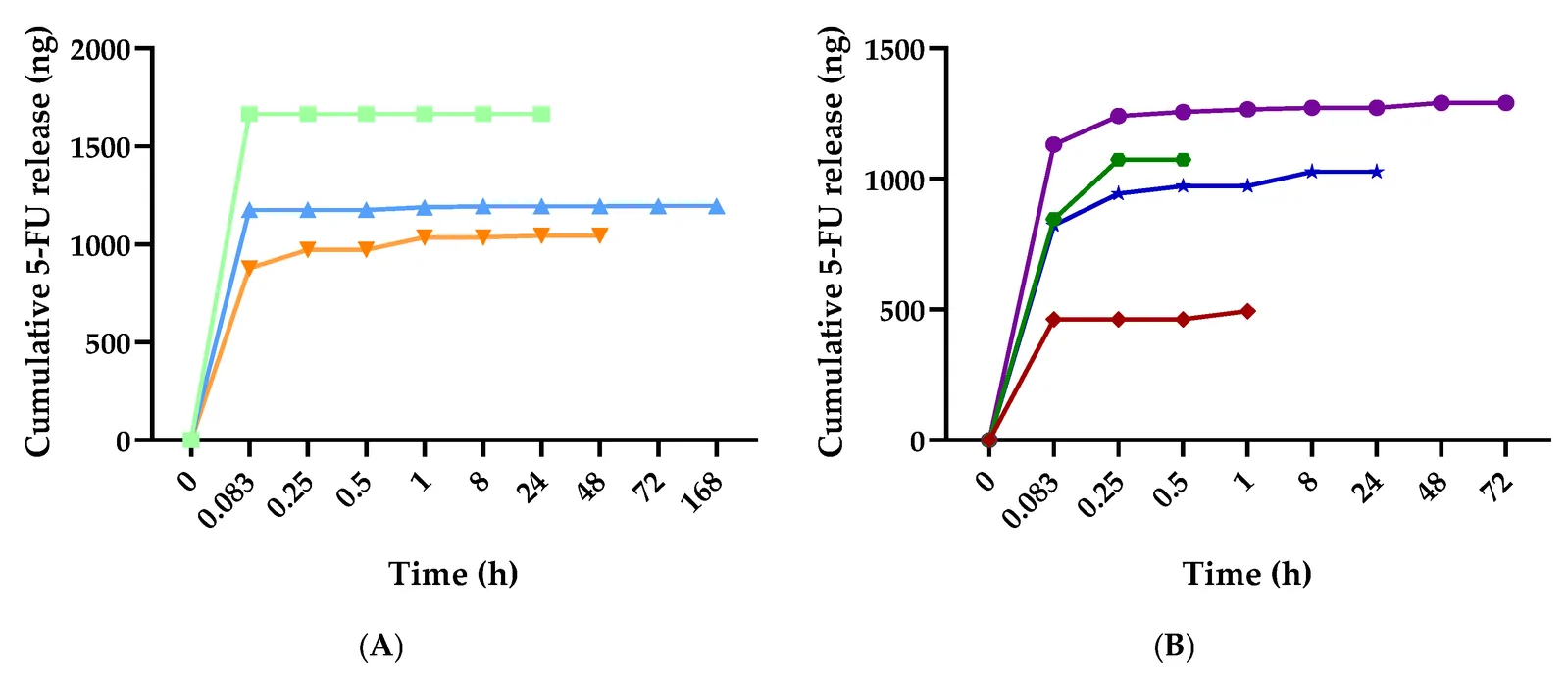
Drug release studies. (**A**) Type A films; (**B**) Type B films. The drug release was assessed by HPLC for three Type A films and four Type B films. In panel B, the purple data series corresponds to a film deposited on a PDMS substrate, while the remaining series in both panels correspond to films deposited on quartz substrates. Each curve represents an independent film preparation; therefore, the release profiles are presented individually rather than mean ± SD, since the number of consecutive reliable quantification points varied among films. A burst release is observed within the first 5 min, followed by a plateau region thereafter.

Drug release studies were carried out as described in the materials and methods section of this paper, and both films, without GO (type A film) and with GO (type B film), were evaluated over time. However, irregular peak shapes and baseline/signal instability, likely associated with instrumental or methodological limitations, prevented the reliable quantification of a few time points. These difficulties were more pronounced in films containing GO, probably due to residual GO in solution affecting the measurements, as GO absorbs in the 200–300 nm range. Therefore, Figure 4 shows only the data up to the last time point with consecutive reliable readings. No interpolation or extrapolation was performed beyond the last consecutive reliable quantification point. Figure 4A shows the data for type A films and Figure 4B shows the data for type B films. Although GO was incorporated with the aim of delaying drug release, in both cases a burst release was observed within the first 5 min, followed by a plateau. Although the type B films had a slight release after the initial 5 min, the concentration was very low. Interestingly, among the tested conditions, the type B deposited on the PDMS substrate showed the most favorable release profile, which may indicate that the molecules might have a greater affinity for this substrate. This PDMS condition was included as an exploratory substrate for the selected GO-containing architecture, given its similarity to the GDD material. The rapid release profile suggests that the drug is weakly retained within the multilayer structure, presumably due to incomplete layer deposition. The lack of a clear delay in release from GO-containing films might also be related to this and indicates that, under the current film architecture, the GO barrier effect of graphene oxide was not sufficient to measurably prevent the initial burst release. Given the burst-dominated release profile and the limited number of consecutive reliable quantification points after the initial release, formal kinetic modeling was not considered appropriate, as it could lead to overinterpretation of the data.

**Figure 5:**
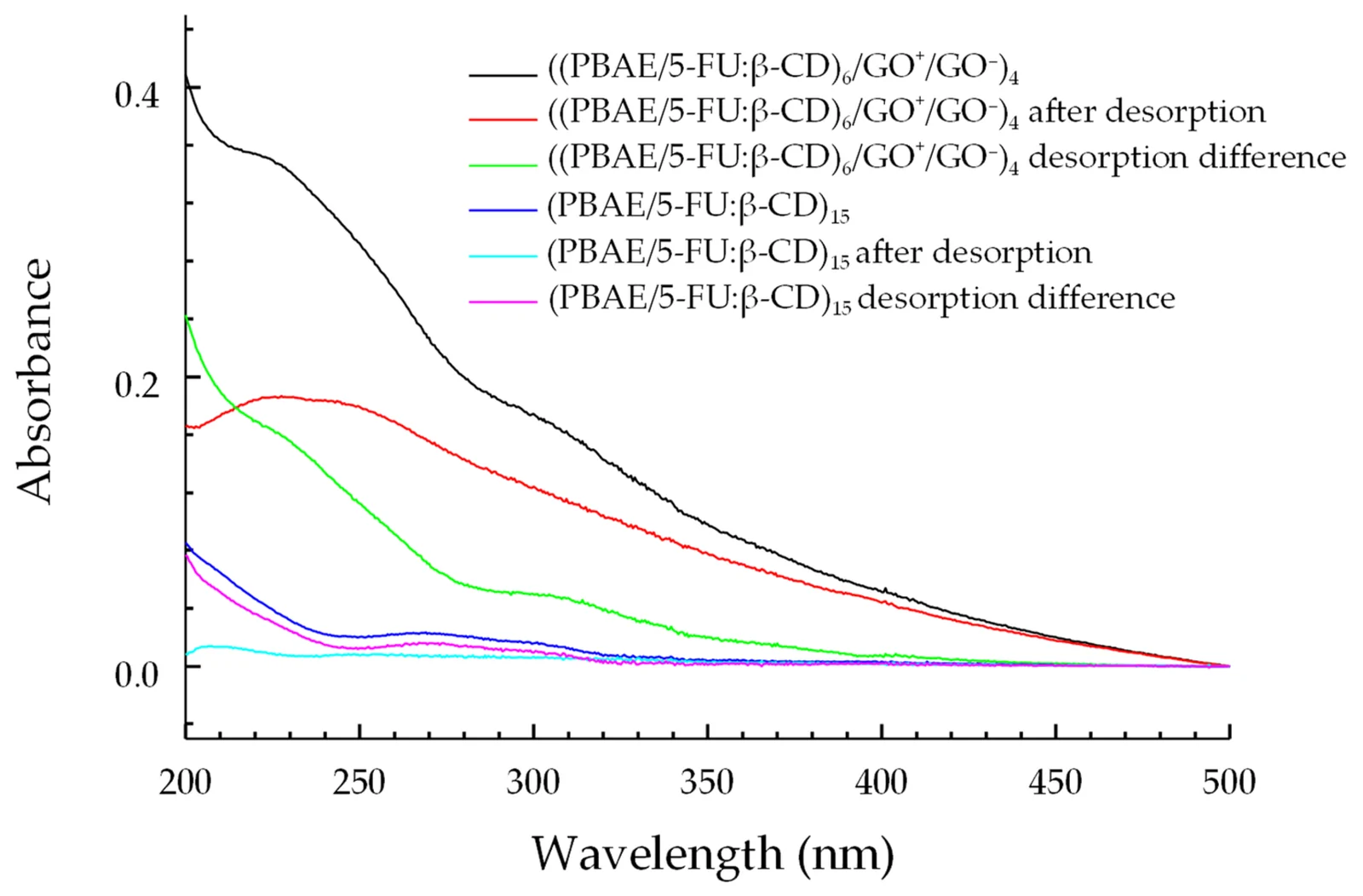
UV-Vis spectra of a representative type A film—(PBAE/5-FU:β-CD)_15_ and type B film—((PBAE/5-FU:β-CD)_6_/GO^+^/GO^−^)_4_ before and after desorption, as well as the graphical representation of the desorption difference spectra.

**Figure A1:**
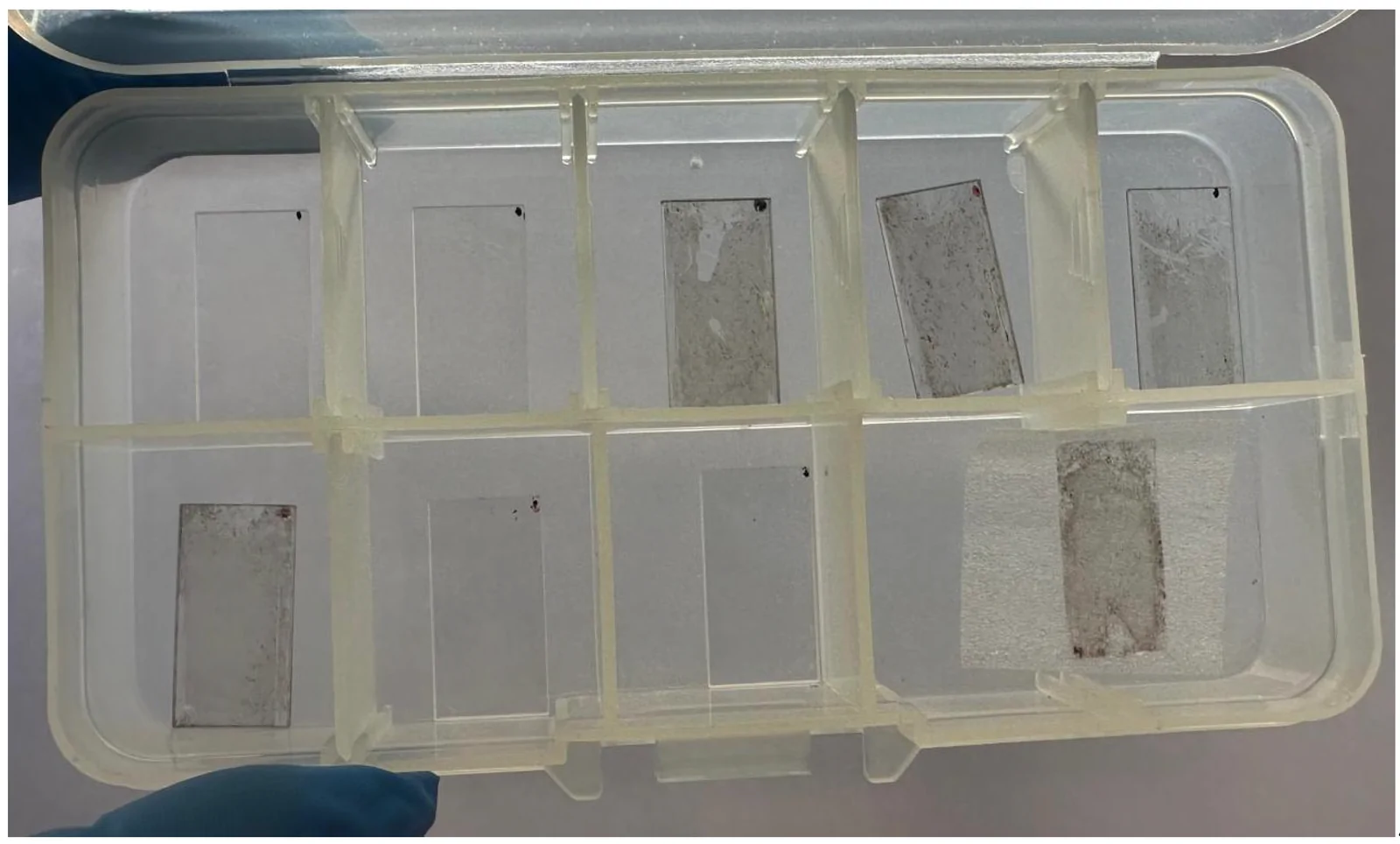
Photo showing type A film—(PBAE/5-FU:β-CD)_15_ and type B film—((PBAE/5-FU:β-CD)_6_/GO^+^/GO^−^)_4_ films after desorption. Films number 1, 2, 7 and 8, counting from the top to the bottom and left to right, are type A films, while the remainder are type B films. All but the last one are in quartz substrates, with the last one being PDMS.

Visual inspection of the films after the drug release study Figure A1, Appendix A, demonstrated that GO was not completely desorbed from the substrate. Taking this into account, the final UV-Vis spectra after desorption were recorded for each of the type A and Type B films deposited on the quartz substrates. Figure 5 shows the UV-Vis spectra of a representative type A film, (PBAE/5-FU:β-CD)_15_, and a representative type B film, ((PBAE/5-FU:β-CD)_6_/GO^+^/GO^−^)_4_, both before and after desorption, as well as the difference spectra for each film representing the material removed during the desorption process (desorption difference spectra). These were calculated by subtracting the post-desorption spectrum from the pre-desorption spectrum in each film.

**Figure 6:**
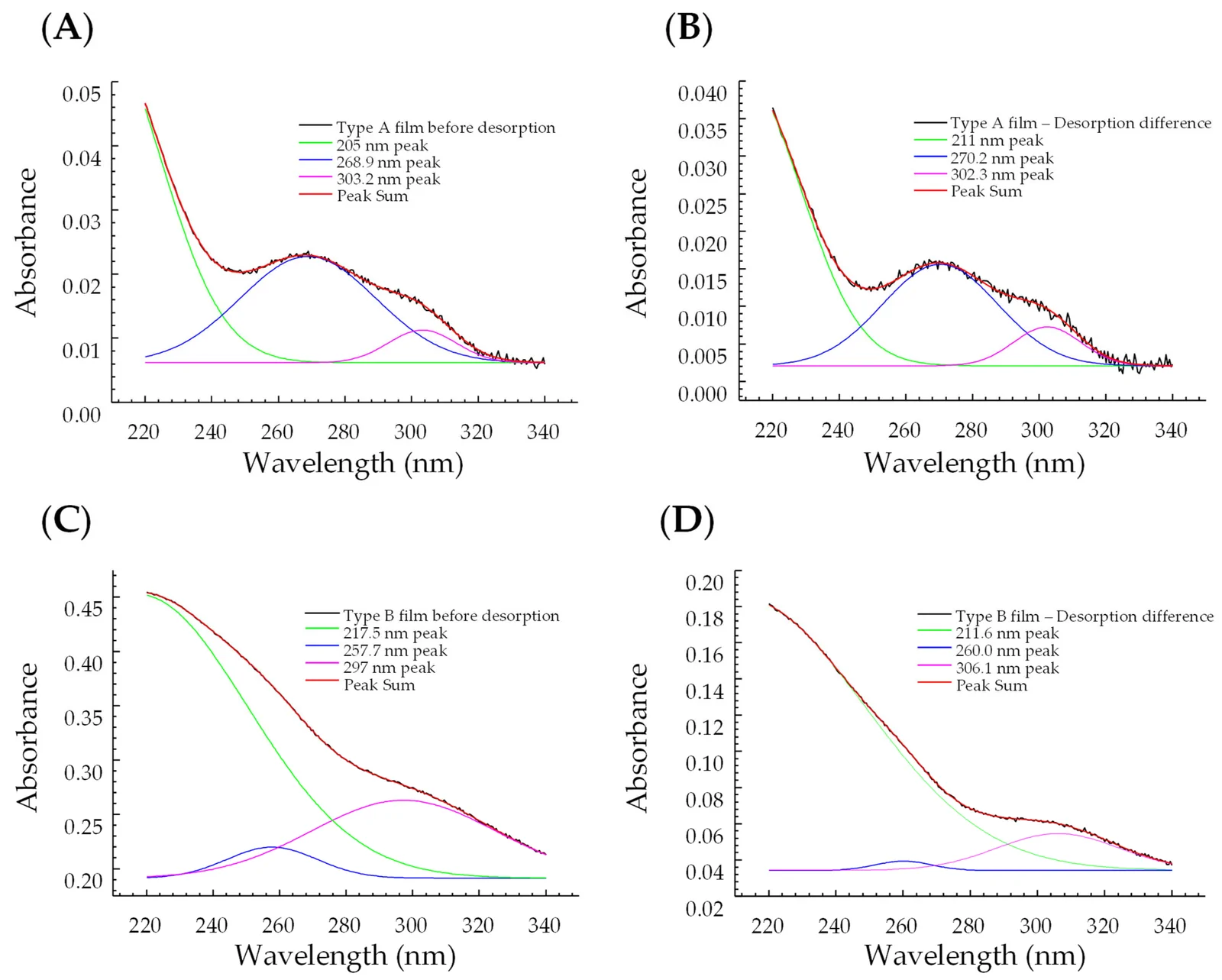
UV-Vis spectra of a representative type A film (PBAE/5-FU:β-CD)_15_ before desorption (**A**) and its desorption difference spectrum (**B**) and of a type B film ((PBAE/5-FU:β-CD)_6_/GO^+^/GO^−^)_4_ before desorption (**C**) and its desorption difference spectrum (**D**). The green, blue, and magenta curves are the fitted Gaussian peaks.

Peak analysis by Gaussian deconvolution was performed on the UV-Vis spectra before desorption and on the desorption difference spectra in each film. This allowed us to identify each peak in the spectrum as presented in Figure 6. Figure 6A,B show the UV-Vis spectra of a (PBAE/5-FU:β-CD)_15_ film before desorption and the respective desorption difference spectra, while Figure 6C,D show the spectra of a (PBAE/5-FU:β-CD)_6_/(GO^+^/GO^−^)_4_ film before desorption and the respective desorption difference spectra. The green, blue, and magenta curves in these figures represent the fitted Gaussian peaks of the spectrum and the red curves are the sum of the peak curves. The peak of interest is centered in the 260 to 270 nm wavelength range, which is associated with 5-FU absorbance and corresponds to the blue curves in the graphs. For reference, Samy et al. measured the UV spectrum of 5-FU in 0.5% acetic acid and found a peak at 265.2 nm, while Machado et al. found a peak near 268 nm when measuring the spectra of 5-FU with varying concentrations of β-CD at pH 7.1 [58,65]. Machado et al. also demonstrated that the 5-FU peak position is pH-dependent [58]. Consequently, the absorbance peak of 5-FU is expected to be influenced by the electrical charges of the other molecules present in the films, and its position may change during the desorption process. Indeed, Figure 6 shows a shift in the position of the 5-FU peak in the spectra associated with the desorbed material. Table 3 summarizes the deconvolution results of the 5-FU peak fitted in the spectra of Figure 6. The values of absorbance at maximum absorbance were not displayed in the table because they varied slightly between samples.

From the analysis of the 5-FU-associated peaks deconvoluted from UV-Vis spectra, before and after desorption, it was possible to estimate a relative 5-FU-associated desorption percentage based on peak height. The average of the peak heights of 5-FU before desorption ($H_{bD}$) and of desorbed material ($H_{D}$) for each type of film is listed in Table 4. The relative percentage of desorbed material (%DM) was calculated using the relation:

(3) $$\%DM=\frac{H_{D}}{H_{bD}}\times 100$$

The calculated %DM listed in Table 4 reveals a higher 5-FU-associated desorption for type A films (71%) than for type B films (28.9%). These values should not be interpreted as absolute percentages of total 5-FU released, since drug loading was not independently determined. Instead, they represent relative spectroscopic values based on 5-FU-associated peak-height ratios supporting the interpretation that GO-containing films retain a larger fraction of the 5-FU-associated signal after desorption.

**Figure 7:**
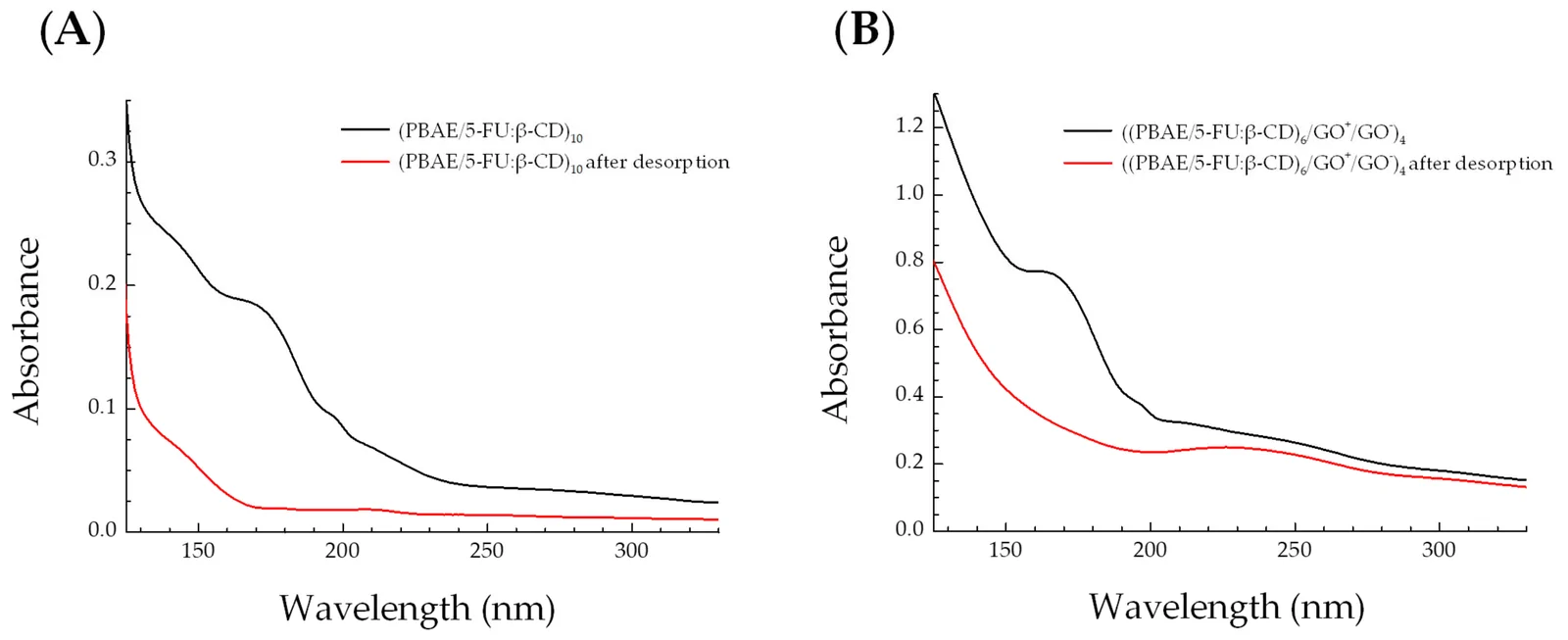
VUV spectra between 120 and 330 nm representing the desorption for 1 min of (**A**) (PBAE/5-FU:β-CD)_10_ and (**B**) ((PBAE/5-FU:β-CD)_6_/GO^+^/GO^−^)_4_ (type B film).

Similar to the evaluation of the UV-Vis adsorption spectra, desorption was also analyzed through VUV spectroscopy as described in the materials and methods section of this paper. Results are represented in Figure 7A,B, which show representative spectra acquired before and after 1 min of desorption in PBS for a type A film, (PBAE/5-FU:β-CD)_10_, and a type B film, ((PBAE/5-FU:β-CD)_6_/GO^+^/GO^−^)_4_, respectively. By analyzing these spectra, it is possible to observe that in films without GO (type A), the VUV signal markedly decreased after desorption, suggesting extensive desorption, whereas in films with GO (type B), the VUV signal remained substantial after 1 min, indicating a greater retention of material on the film structure and suggesting that a considerable part of the drug-loaded multilayer structure remains. These observations are consistent with UV-Vis desorption analysis and support the interpretation that even though desorption occurs in both films, GO contributes to the retention of 5-FU in the film after short-term desorption. It is worth noting that this spectroscopic evidence does not contradict the HPLC release results, since HPLC quantifies 5-FU released into solution whereas UV-Vis and VUV assess residual 5-FU-associated signals. Therefore, GO-containing films may retain part of the drug-loaded multilayer structure while still showing an initial burst release of the readily available 5-FU fraction.

**Figure 8:**
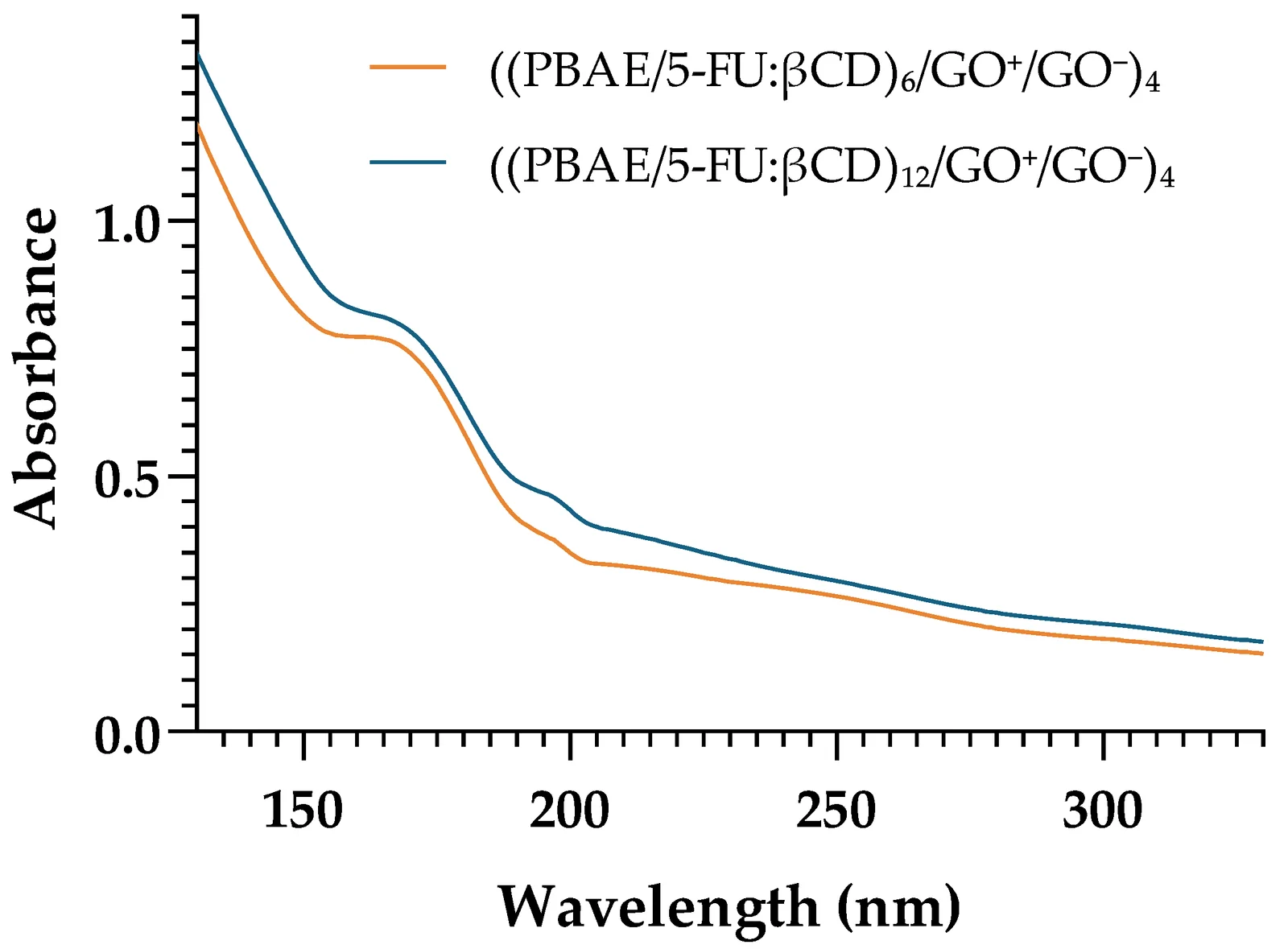
VUV spectra between 130 and 330 nm comparing type B films prepared with either 6 or 12 PBAE/5-FU:β-CD bilayers between each GO^+^/GO^−^ block: ((PBAE/5-FU:β-CD)_6_/GO^+^/GO^−^)_4_ and ((PBAE/5-FU:β-CD)_12_/GO^+^/GO^−^)_4_, respectively.

Additional VUV measurements were performed to compare type B films prepared with either 6 or 12 PBAE/5-FU:β-CD bilayers between each GO^+^/GO^−^ block (Figure 8). Although the film with 12 drug-loaded bilayers showed a slightly higher absorbance signal, this increase was not proportional to the doubling of the number of PBAE/5-FU:β-CD bilayers. This suggests that increasing the number of drug-loaded layers between GO insertions does not lead to a proportional increase in the VUV absorbance signal, possibly due to film saturation or limited incorporation efficiency.

### 2.3. Cell Cycle Arrest

To confirm whether β-CD complexation preserved the biological activity of 5-FU, cell cycle distribution was assessed by flow cytometry after 24 h of exposure. This analysis was performed given that 5-FU is expected to interfere with DNA synthesis and induce S-phase arrest in actively proliferating cells [49].

Three conditions, each at two dose levels, were tested: 5-FU (0.3 mg and 0.075 mg), 5-FU:β-CD complexes (containing 0.3 mg and 0.075 mg of 5-FU) and β-CD (amounts equivalent to those used to complex 0.3 mg and 0.075 mg of 5-FU, corresponding to approximately 2.65 mg and 0.66 mg of β-CD, respectively). Since the complex was prepared at a 1:1 molar ratio and β-CD has a molecular weight approximately 8.82-fold higher than 5-FU, the corresponding β-CD mass was proportionally higher. Therefore, the β-CD controls were prepared using the amount of β-CD equivalent to that present in the corresponding 5-FU:β-CD complexes. The 0.3 mg dose was diluted in 1 mL, while the 0.075 mg dose was diluted in 250 µL of PBS. Cells were processed for cell cycle analysis by flow cytometry 24 h after adding the corresponding volume of each condition to each well [49,57,66,67].

**Figure 9:**
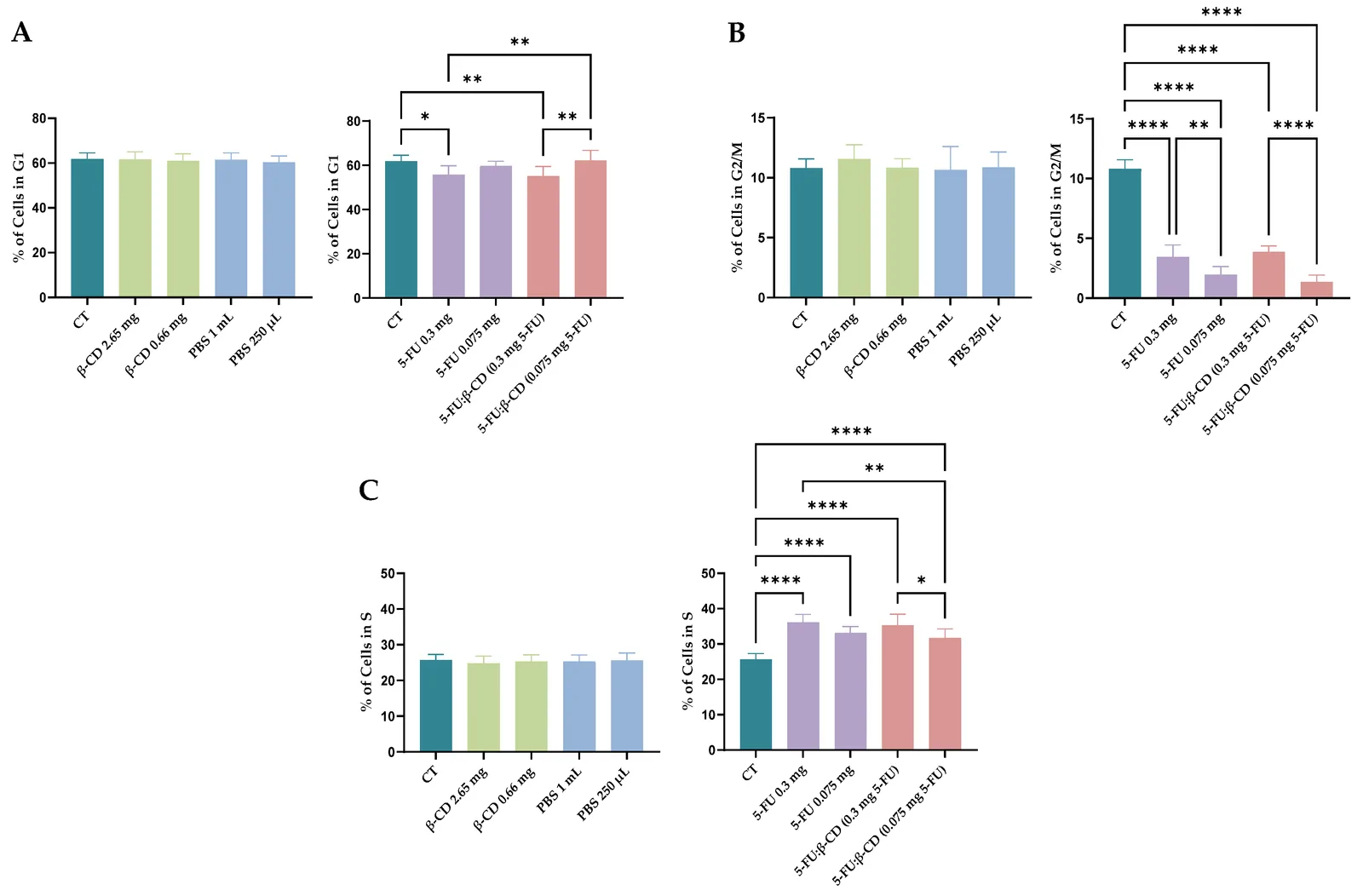
Effect of 5-FU on the cell cycle distribution. (**A**) G1-phase distribution; (**B**) G2/M-phase distribution; (**C**) S-phase distribution. In each panel, control conditions (CT, β-CD, and PBS controls) and 5-FU/5-FU:β-CD treatment conditions are presented separately. Flow cytometry was used to analyze the effect of two different dose levels of 5-FU alone or complexed with β-CD (5-FU:β-CD). 5-FU induced cell cycle arrest in the S phase, with a stronger effect at the higher dose, while β-CD controls did not appear to affect cell cycle distribution. The control (CT) corresponds to untreated cells, and PBS was used as the dissolution medium for all conditions. For clarity, β-CD/PBS controls and 5-FU/5-FU:β-CD conditions are presented separately. β-CD controls were prepared using the amount of β-CD equivalent to that present in the corresponding 5-FU:β-CD complexes. Data are expressed as the percentage of cells in each cell cycle phase. A total of 30,000 cells were analyzed per experiment, with *n* = 3 independent experiments. Statistical significance was assessed by ordinary one-way ANOVA followed by Tukey’s multiple comparisons test. Adjusted *p* values indicate statistical significance: *p* < 0.0001 (****); *p* < 0.0021 (**); *p* < 0.0332 (*).

As shown in Figure 9, treatment with 5-FU increased the percentage of cells in S phase, consistent with the expected mechanism of action of 5-FU as an inhibitor of DNA synthesis [66,67]. This effect was more pronounced at the higher dose and was also observed when 5-FU was complexed with β-CD, indicating that complexation did not impair the biological activity of the drug [57]. In contrast, β-CD alone did not substantially alter cell cycle distribution compared with the untreated and PBS controls, supporting its suitability as a carrier in this system.

## 3. Discussion

The present work aimed to develop a spray-assisted LbL DDS for localized application in GDD surgery, targeting the suppression of fibroblast proliferation. The results demonstrate that the proposed fabrication method enables the formation of multilayer films with consistent and sequential growth, as confirmed by UV-Vis spectroscopy. Type A films exhibited lower variability and more reproducible growth profiles than type B films, potentially due to the additional complexity introduced by the insertion of GO layers and the manual spray deposition process.

In addition to UV-Vis analysis, VUV spectroscopy provided further insight into the internal structure and loading behavior of the films. While both techniques confirmed sequential film growth, VUV data revealed that increasing the number of drug-loaded layers between graphene insertions does not result in a proportional increase in overall drug content. This suggests the existence of a saturation effect within the multilayer architecture, presumably associated with limited available binding sites or inefficient layer adsorption.

Despite the successful incorporation of GO as a potential diffusion barrier, drug release studies revealed a rapid burst release within the first few minutes, followed by a plateau, for both film types. This behavior suggests that a fraction of the incorporated 5-FU is weakly retained within the multilayer structure, which can be explained by its low molecular weight, high polarity, and hydrophilic nature, limiting its interaction with the relatively hydrophobic PBAE matrix. Although β-cyclodextrin complexation was employed to enhance drug incorporation, this strategy alone appears insufficient to significantly modulate release kinetics under the tested conditions.

From a practical standpoint, the films demonstrated good shelf stability, maintaining their structural integrity and performance after storage for up to one month prior to the drug release studies, which supports their potential for practical handling and preoperative preparation in clinical settings.

HPLC measurements suggested that the incorporation of GO did not result in a substantial delay in drug release, contrary to what would be expected from its role as a diffusion barrier. However, UV-Vis and VUV analyses demonstrated that GO-containing films retained a significant fraction of 5-FU after desorption, suggesting partial drug retention within the multilayer structure. This suggests that GO incorporation alone is not sufficient to modulate release kinetics under these deposition conditions. Possible explanations include incomplete surface coverage, structural heterogeneity of the films, or partial entrapment of the drug in the film structure. Additionally, visual inspection of the substrates indicated that GO layers remain present even after prolonged desorption.

From a biological perspective, cell cycle analysis confirmed that 5-FU retained its activity after complexation with β-CD, inducing S-phase arrest in a concentration-dependent manner. This is consistent with the known mechanism of action of 5-FU and supports the suitability of the complexed drug for incorporation into DDS platforms. The absence of effects from β-CD alone further confirms its biocompatibility in this context.

Overall, while the proposed system demonstrates feasibility for film fabrication and drug incorporation, and may contribute to more standardized treatment approaches, the lack of sustained release highlights the need for further optimization. Future work should focus on improving drug retention within the films, potentially through increased film density, alternative barrier strategies, or the use of additional crosslinking mechanisms. Furthermore, optimizing the deposition technique to reduce variability and enhance layer uniformity may contribute to more predictable and controlled release profiles. These improvements are essential for the translation of this approach into clinically relevant coatings for glaucoma surgical devices.

## 4. Materials and Methods

### 4.1. Reagents

PBAE was synthesized using the method described by Lynn et al. [55]. Briefly, 1,4-butanediol diacrylate (0.750 g, 0.714 mL, 3.78 mmol; 99% purity, Alfa Aesar GmbH & Co., KG, Karlsruhe, Germany; CAS 1070-70-8) and 4,4′-trimethylenedipiperidine (3.78 mmol; 97% purity, Sigma-Aldrich, St. Louis, MO, USA; CAS 16898-52-5) were separately dissolved in distilled tetrahydrofuran (THF) (Alfa Aesar GmbH & Co. KG, Karlsruhe, Germany; 47122) (5 mL each). The diamine solution was added dropwise to the diacrylate solution; the mixture was stirred with a Teflon-coated stir bar, sealed under a Teflon-lined cap, and heated at 50 °C for 48 h. After cooling, the reaction mixture was precipitated into vigorously stirred diethyl ether (Sigma-Aldrich, St. Louis, MO, USA; 179272). The resulting polymer was collected by vacuum filtration (Büchner funnel) and dried under vacuum overnight. The final PBAE structure (Table 5) was confirmed by ^1^H and ^13^C nuclear magnetic resonance (NMR) spectroscopy (Bruker, Billerica, MA, USA; Avance II 300 MHz).

The 5-FU and β-CD were acquired from Sigma-Aldrich (St. Louis, MO, USA; F6627 and C2485, respectively) (see Table 5 for chemical structure).

GO^−^ was acquired from Graphenea (San Sebastián, Spain; GOCD21057). GO^+^ was synthesized from GO^−^ according to the following: 50 mL of GO^−^ was mixed with 0.625 g of N-ethyl-N’-(3-dimethylaminopropyl) carbodiimide methiodide (EDC) (Tokyo Chemical Industry Co., Ltd. (TCI), Tokyo, Japan; D5334) and 5 mL of ethylenediamine (Sigma-Aldrich, St. Louis, MO, USA; E26266). The solution was left stirring for 24 h at room temperature (RT). After this, the solution was purified using a cellulose membrane (Sigma-Aldrich, St. Louis, MO, USA; D9527) previously washed and submerged in distilled water for 24 h. For the purification, the cellulose membrane containing the solution was submerged in distilled water changed every 24 h, until the pH of the water reached 6. The pH of the GO^+^ was then adjusted to 5.0 with glacial acetic acid (Merck KGaA, Darmstadt, Germany; 1.00063.2511) [68]. See Table 5 for GO^+^ and GO^−^ chemical structures.

**Table 5:** Chemical structures of the materials used in the preparation of the thin films. The 5-FU structure was obtained/adapted from PubChem; the PBAE structure was drawn by the authors using RDKit (2026.03.3) in Google Colab based on the cited reference; β-CD and GO^−^ structures were reproduced from the cited references under the Creative Commons Attribution License (CC BY 4.0) and the GO^+^ structure was adapted from a master’s thesis with permission.

Molecule	Chemical Structure	Acronym
5-fluorouracil [69]	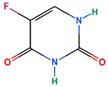	5-FU
Poly β-amino ester [55]	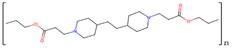	PBAE
β-cyclodextrin [70]	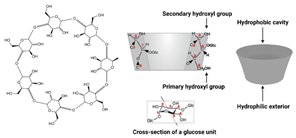	β-CD
Positively Charged Graphene Oxide [71]	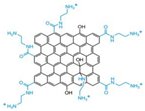	GO^+^
Negatively Charged Graphene Oxide [72]	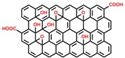	GO^−^

### 4.2. Complexation 5-FU:β-CD

A sodium acetate (VWR Chemicals, Leuven, Belgium; 27652.260) solution at pH 5.5, adjusted with acetic acid, was prepared by dissolving 0.433 g of sodium acetate in distilled water for a final concentration of approximately 0.106 M.

5-FU and β-CD were complexed by dissolving them in the aforementioned sodium acetate buffer and stirring at a 1:1 molar ratio for 24 h at room temperature. Since the polymer β-CD is used for this calculation, the molecular weight of the β-CD monomer was considered. To prepare 10 mL of solution, 0.06 g of β-CD and 0.0068 g of 5-FU were used, yielding a final concentration of 5.2 mM for each.

This protocol was previously used and optimized by us using brimonidine [54].

### 4.3. Layer-by-Layer Film Deposition

For film deposition, three types of substrates were used. Crystal Quartz substrates (24 mm × 12.5 mm × 1 mm, Crystran Ltd., Poole, UK), calcium fluoride substrates (CaF_2_, 20 mm diameter, Crystran Ltd., Poole, UK) and polydimethylsiloxane (PDMS) substrates (Dow, Midland, MI, USA; Sylgard™ 184) were kindly provided by Monica Machado from the Organic Electronics group (Telecommunications Institute). Quartz substrates were chosen to allow film growth tracking with UV-Vis; CaF_2_ substrates were chosen to allow tracking using Fourier-transform infrared spectroscopy (FTIR) and PDMS substrates were chosen for their similarity with the glaucoma drainage device valve.

Quartz substrates were thoroughly cleaned before film deposition with piranha solution (1:1, H_2_O_2_:H_2_SO_4_) (H_2_O_2_ 33%, VWR Chemicals, Leuven, Belgium; 23613.297; H_2_SO_4_, Fisher Chemical, Loughborough, UK; S/9240/PB17). In addition to cleaning, the piranha solution also imparts a negative charge to the substrate, promoting the adhesion of the first, positively charged PBAE layer. After being washed with piranha solution, the substrates were thoroughly rinsed with water and dried with a nitrogen (N_2_) flow (Sociedade Portuguesa do Ar Líquido (Air Liquide), Algés, Portugal; Alphagaz^TM^ 1) [53,73,74,75].

Furthermore, before deposition, all substrates were functionalized by oxygen plasma treatment for 3 min (Harrick Plasma, Ithaca, NY, USA; PDC-002-CE), which oxidizes and functionalizes the surface, generating negatively charged oxygen-containing groups [76].

PBAE was freshly prepared every 3.5 h due to its high degradation rate when in aqueous solution, by dissolving 0.0237 g of PBAE in sodium acetate solution for a final approximate monomer-equivalent concentration of 8 mM [55].

For the spray deposition, perfume atomizers (Equivalenza Retail S.L.U., L’Hospitalet de Llobregat, Barcelona, Spain) and 3D-printed supports were used to guarantee that the distance (6 cm) and placement of the substrate were the same every time.

Two types of films were produced: films without GO with the composition (PBAE/5-FU:β-CD)_15_ (Film type A) and films with GO with the composition ((PBAE/5-FU:β-CD)_6_/GO^+^/GO^−^)_4_ (Film type B). Schemes of these films are displayed in Figure 1 in the introduction of this paper. For the deposition, 5 spray pushes for PBAE and GO and 6 spray pushes for 5-FU:β-CD were used. Between each layer deposition, the substrate was dried with an N_2_ flow and a UV-Vis spectrum between 200 and 500 nm was acquired (JASCO Corporation, Hachioji, Tokyo, Japan; V-730) for each 5-FU:β-CD and GO layer. No measurements were taken for the PBAE layers, as this polymer does not exhibit absorbance within this spectral range. In addition, other formulations were explored during method optimization, including halving the 5-FU:β-CD concentration, introducing sodium acetate washes between each layer, varying the number of spray applications per layer, and using spin coating instead of the spray-assisted technique. None of these approaches proved effective; ultimately, the previously described technique was selected.

For VUV measurements, a third type of film was produced with the composition (PBAE/5-FU:β-CD)_10_ (Film type C). In this case, instead of sequential measurements as the film was being deposited, several films were produced with the desired number of layers and only one measurement (on the final layer) was made per film. The plots were then generated according to this. Measurements were performed at the AU-UV beamline of the ASTRID2 synchrotron radiation facility (ISA, Department of Physics and Astronomy, Aarhus University, Aarhus, Denmark). UV/VUV transmission spectra were acquired in the 110–330 nm range using a monochromator equipped with a grating system. The transmitted intensity was detected using a photomultiplier tube (PMT) under vacuum conditions. Reference measurements were performed using clean CaF_2_ windows. Data acquisition and experimental control were performed using locally developed software.

### 4.4. Dissolution Study/Drug Release

For the drug release study, films were produced according to the aforementioned methodology on substrates with dimensions 24 mm × 12.5 mm, with the coating deposited on only one of the faces of the substrate, corresponding to an approximate area of 3 cm^2^. The coated substrates were then transferred to airtight vials, with the films facing up and submerged in 2 mL of PBS at pH 7.2 (Thermo Fisher Scientific, Waltham, MA, USA; 70013032). The vials were then incubated at 37 °C under constant orbital agitation (100 rpm). At each predetermined time point, 1 mL of the dissolution media was collected for subsequent HPLC analysis and 1 mL of fresh PBS was added. After the final time point, a new UV-Vis reading was performed, except for the film deposited on the PDMS substrate.

### 4.5. HPLC Measurements

Sample analysis was carried out using a Waters Alliance 2695 HPLC system (Waters Corporation, Milford, MA, USA) equipped with a Waters 2996 photodiode array (PDA) (Waters Corporation, Milford, MA, USA) detector. A wavelength scan was performed between 190 and 400 nm, and each run lasted 7 min. The column used was a Symmetry C18 (Waters Corporation, Milford, MA, USA; 250 × 4.6 mm, 5 µm particle size), maintained at 30 °C. The mobile-phase flow rate was 1.0 mL/min and the injection volume was 50 µL. Peak analysis was performed using Empower 3 software (Waters Corporation, Milford, MA, USA). The 5-FU peak has its λ_max_ around 266 nm, and its retention time is approximately 3.5 min for the following running conditions: running buffer was PBS (0.1752 g of sodium dihydrogen phosphate (NaH_2_PO_4_, Sigma-Aldrich, St. Louis, MO, USA; S0751) and 3.6 g of disodium hydrogen phosphate (Na_2_HPO_4_, Merck KGaA, Darmstadt, Germany; 1.06586.0500) per liter, pH ≈ 5.2, adjusted with hydrochloric acid (HCl, VWR Chemicals, Leuven, Belgium; 20252.244)) with 7% acetonitrile (ACN, Fisher Chemical, Loughborough, UK; A/0627/17), premixed before injection and 7% of ACN was added to each sample before the run according to the formula.

ACN% = *X*/(sample volume + *X*) (4)

Samples were filtered prior to analysis using polyethersulfone (PES) syringe filters with a 0.22 µm pore size (Labbox Labware S.L., Premià de Dalt, Barcelona, Spain; SFPE-12E-100).

Quantification of 5-FU was performed using external calibration curves prepared in PBS with 7% ACN. Due to the concentration range of the samples, two calibration ranges were used: a low range from 6.563 to 52.5 ng/mL, including a blank at 0 ng/mL, and a high range from 52.5 to 840 ng/mL. The coefficients of determination were *R*^2^ = 0.9985 for the low-range calibration curve and *R*^2^ = 0.996 for the high-range calibration curve. The practical limit of quantification was defined as the lowest non-zero calibration standard included in the accepted calibration curve, corresponding to 6.563 ng/mL.

### 4.6. Cell Cycle Arrest—Cell Culture

Human, spontaneously transformed, retinal pigment epithelial cells (D407), kindly provided by Dr. Jean Bennett (University of Pennsylvania, Philadelphia, PA, USA), were used to study whether β-CD affected the effect of 5-FU on the cell cycle. Cells were cultured in HyClone™ Dulbecco’s Modified Eagle Medium (DMEM) with high glucose (Cytiva, Marlborough, MA, USA; SH30285.01) at 37 °C, 5% carbon dioxide (CO_2_). Cells were seeded at a density of 150,000 cells/well in 6-well plates and after growing for 24 h, the experiment began. Two types of controls were used: cells without any treatment and cells with PBS (1 mL and 250 µL). Three conditions, each at two dose levels, were tested: 5-FU (0.3 mg and 0.075 mg), 5-FU:β-CD complexes (containing 0.3 mg and 0.075 mg of 5-FU) and β-CD (amounts equivalent to those used to complex 0.3 mg and 0.075 mg of 5-FU, corresponding to approximately 2.65 mg and 0.66 mg of β-CD, respectively). Since the complex was prepared at a 1:1 molar ratio and β-CD has a molecular weight approximately 8.82-fold higher than 5-FU, the corresponding β-CD mass was proportionally higher. Therefore, the β-CD controls were prepared using the amount of β-CD equivalent to that present in the corresponding 5-FU:β-CD complexes. The 0.3 mg dose was diluted in 1 mL, while the 0.075 mg dose was diluted in 250 µL of PBS. Cells were processed for cell cycle analysis through flow cytometry 24 h after adding the corresponding volume of each condition to each well.

### 4.7. Cell Cycle Arrest—Flow Cytometry

For flow cytometry cell cycle analysis, the cells were washed once with PBS, trypsinized (Biowest, Nuaillé, France; L0931-100) and centrifuged at 440 *g* for 1 min, followed by two more PBS washes. After that, cells were vigorously resuspended in 500 µL of PBS at 4 °C and fixed with 4.5 mL of 70% ethanol at −20 °C, added dropwise while vortexing for at least 2 h. Next, cells were centrifuged at 400 *g* for 5 min and washed three times with PBS and resuspended in 100 µL of PBS with 100 µg/mL of ribonuclease A (RNase A) (Qiagen, Hilden, Germany; 1072590) and 50 µg/mL of propidium iodide (PI) (Invitrogen/Thermo Fisher Scientific, Waltham, MA, USA; P3566). Cells were run through the FACSCanto II flow cytometer (BD Biosciences, Franklin Lakes, NJ, USA) with FACSDiva v.8.0.3 software (BD Biosciences, Franklin Lakes, NJ, USA) 30 min after the resuspension.

### 4.8. Analysis Software

Data resulting from film growth and drug release experiments were analyzed using GraphPad Prism 9 (GraphPad Software, Boston, MA, USA) and OriginPro 8.5 (OriginLab Corporation, Northampton, MA, USA). Data resulting from cell cycle studies were analyzed using FlowJo 10.10.1 (FlowJo LLC, Ashland, OR, USA) and Microsoft Excel for Office 365 (Microsoft Corporation, Redmond, WA, USA). Cell cycle distributions were determined from DNA content using the Watson Pragmatic model.

## 5. Conclusions

In this work, we successfully developed an LbL DDS using the spray-assisted technique. The films exhibited consistent growth and drug incorporation (confirmed by UV-Vis and VUV spectroscopy), with type A films showing lower variability compared with type B films. Despite the incorporation of GO as a diffusion barrier, both systems displayed a rapid burst release profile, suggesting limited control over sustained drug delivery under the tested conditions. Furthermore, VUV analysis revealed saturation behavior in drug loading, indicating structural limitations in the multilayer architecture. Notably, β-CD complexation did not compromise the biological activity of 5-FU, which effectively induced S-phase cell cycle arrest in a concentration-dependent manner. These findings demonstrate the feasibility of the proposed system while highlighting the need for further optimization to achieve controlled and prolonged drug release before clinical applicability can be considered.

## Figures and Tables

**Table 1:** Block increments for an individual type B film.

Δ*A* (6–1) = *A*_6_ − *A*_1_	0.0084 ± 0.0002
Δ*A* (12–7) = *A*_12_ − *A*_7_	0.0158 ± 0.0002
Δ*A* (18–13) = *A*_18_ − *A*_13_	0.0123 ± 0.0002
Δ*A* (24–19) = *A*_24_ − *A*_19_	0.0170 ± 0.0002

**Table 2:** Area under the curve (AUC) and relative standard deviation (RSD) for type A and B films.

Type of Films	AUC	RSD (%)
A	0.173 ± 0.017	9.6
B	4.4 ± 1.1	25.5
B without graphene offset	0.83 ± 0.14	16.6

**Table 3:** Fitting characteristics of 5-FU peak for type A and B films. PP = Peak Position.

Type of Films	PP Before Desorption (nm)	Peak Width Before Desorption	*R* ^2^	PP Desorbed Material (nm)	Peak Width Desorbed Material	*R* ^2^
A	268.9 ± 0.3	41 ± 2	0.99888	270.2 ± 0.4	35 ± 2	0.99631
B	257.7 ± 0.7	17 ± 2	0.99988	260.0 ± 0.3	17 ± 2	0.99988

**Table 4:** Relative spectroscopic desorption analysis of 5-FU for type A and B films, where $H_{bD}$ is the mean value of the peak height of 5-FU before desorption, $H_{D}\;$ is the mean value of the 5-FU peak height of desorbed material and %DM is the relative percentage of desorbed material calculated from peak-height ratios.

Type of Films	$H_{bD}$	$H_{D}$	%DM
A	0.0134 ± 0.0040	0.0097 ± 0.0040	71 ± 15
B	0.0353 ± 0.0097	0.0088 ± 0.0015	28.9 ± 4.7

## Data Availability

The data supporting the findings of this study are available within the article. Additional data are available from the corresponding author upon reasonable request.

## Abbreviations

The following abbreviations are used in this manuscript:

ACN	Acetonitrile
AUC	Area Under the Curve
β-CD	β-Cyclodextrin
CAS	Chemical Abstracts Service
CaF_2_	Calcium Fluoride
CI	Confidence Interval
CO_2_	Carbon Dioxide
CV	Coefficient of Variation
DDS	Drug Delivery System
DHEA-S	Dehydroepiandrosterone Sulfate
DHED	10β,17β-Dihydroxyestra-1,4-dien-3-one
DM	Desorbed Material
DMEM	Dulbecco’s Modified Eagle Medium
DNA	Deoxyribonucleic Acid
EDC	N-ethyl-N’-(3-dimethylaminopropyl) carbodiimide methiodide
FACS	Fluorescence-Activated Cell Sorting
FTIR	Fourier-Transform Infrared Spectroscopy
GO	Graphene Oxide
GO^+^	Positively Charged Graphene Oxide
GO^−^	Negatively Charged Graphene Oxide
GDD	Glaucoma Drainage Device
HCl	Hydrochloric Acid
H_2_O_2_	Hydrogen Peroxide
HPLC	High-Performance Liquid Chromatography
H_2_SO_4_	Sulfuric Acid
IOP	Intraocular Pressure
LbL	Layer-by-Layer
MIGS	Minimally Invasive Glaucoma Surgery
MMC	Mitomycin C
N_2_	Nitrogen
NaH_2_PO_4_	Sodium Dihydrogen Phosphate
Na_2_HPO_4_	Disodium Hydrogen Phosphate
NMR	Nuclear Magnetic Resonance
PACG	Primary Angle-Closure Glaucoma
PES	Polyethersulfone
PMT	Photomultiplier Tube
PBS	Phosphate Buffered Saline
PBAE	Poly(β-amino ester)
PDA	Photodiode Array
PDMS	Polydimethylsiloxane
PI	Propidium Iodide
PP	Peak Position
POAG	Primary Open-Angle Glaucoma
ROCK	Rho-Associated Protein Kinase
RSD	Relative Standard Deviation
RNase	Ribonuclease
RT	Room Temperature
SD	Standard Deviation
THF	Tetrahydrofuran
UV-Vis	Ultraviolet-Visible Spectroscopy
VUV	Vacuum Ultraviolet Spectroscopy
5-FU	5-Fluorouracil

## Appendix A

Visual inspection of the films after the drug release study: Figure A1 clearly shows that GO is not being completely desorbed from the substrate during the release assay.
